# Efficient Methods of Utilizing Multi-SBAS Corrections in Multi-GNSS Positioning

**DOI:** 10.3390/s20010256

**Published:** 2020-01-01

**Authors:** Kwi Woo Park, Jong-Il Park, Chansik Park

**Affiliations:** 1 Department of Control and Robotics Engineering, Chungbuk National University, Cheongju 28644, Korea; zumbox@chungbuk.ac.kr (K.W.P.); whddlf915@naver.com (J.-I.P.); 2 Navcours Co. Ltd., Daejeon 34014, Korea

**Keywords:** GNSS, SBAS, multi-SBAS correction, multi-GNSS positioning, covariance analysis

## Abstract

Various combining methods have been proposed to utilize multi-satellite-based augmentation system (SBAS) correction to provide accurate position in the global navigation satellite system (GNSS) receiver. However, the proposed methods have not been objectively compared and analyzed, making it difficult to know which ones are effective for multi-GNSS positioning. This paper presents efficient methods of combining multi-SBAS corrections in multi-GNSS positioning by comparing three methods: correction domain integration, measurement domain integration, and position domain integration. The performance of the three methods were analyzed through a covariance analysis that was expanded to multi-GNSS and multi-SBAS. Then, the results were verified by experiments using real measurements and corrections. Furthermore, implementation issues, such as computational complexity, availability, and flexibility, are analyzed. As a result, three methods had the same precision, but different complexity, availability, and flexibility. These results will be important guidelines to design, implement, and analyze navigation systems based on multi-GNSS with multi-SBAS corrections.

## 1. Introduction

Global navigation satellite systems (GNSS) have become a major navigation and positioning means in many areas, but the horizontal position accuracy of a standalone GNSS is known to be at least 5.0 m with a 95% confidence level [1]. To improve position accuracy, the receiver has been designed to receive multi-GNSS signals from multi-frequencies and multi-constellation GNSS [2,3,4]. In a multi-GNSS receiver, not only is the global positioning system (GPS) used, but also the Russian global navigation satellites system (GLONASS), the Beidou navigation satellite system (BDS), and Galileo are used to increase the accuracy and reliability of the position [2]. In addition, signal availability can be improved because the probability of receiving more than four satellite signals is improved by using multi-GNSS even in an urban canyon [3]. By using multi-frequencies, accuracy can be further improved by eliminating the ionospheric delay contained in the pseudo-range measurement [4].

Another way to improve the accuracy, precision, and integrity of a GNSS is the use of a differential GNSS (DGNSS). The accuracy and precision of a DGNSS is improved through the elimination of common errors, such as ephemeris error, satellite clock error, and ionospheric and tropospheric delay, using the corrections from reference stations located at known positions. Real-time kinematic (RTK) positioning is a form of DGNSS that uses carrier phase measurements and achieves centimeter-level precision [5,6].

A satellite-based augmentation system (SBAS) uses a geostationary satellite to provide the spatial corrections for a wide area. The fast, long-term, and ionospheric corrections are broadcasted to compensate for pseudo-range, satellite ephemeris, and ionospheric delay. Besides these corrections, SBAS provides degradation parameters, such as fast correction degradation factor, grid ionospheric vertical error indicator, and clock-ephemeris covariance matrix [7,8]. Because the corrections and degradation parameters are broadcast by a GPS-like L1 (1575.42 MHz) signal, the accuracy, precision, and integrity of the GNSS receiver can be further improved compared to when a standalone receiver without an external communication link is used. In [9], SBAS was added to an integrated navigation system to improve the integrity of the vehicle navigation. In [10], SBAS correction was used to enhance the quality of satellite orbits and clock information in real-time single-frequency precise point positioning.

Currently, many SBASs, such as the U.S. Wide Area Augmentation System (WAAS), European Geostationary Navigation Overlay System (EGNOS), Japanese Multi-Functional Satellite Augmentation System (MSAS), Indian GPS and Geostationary equatorial orbit Augmented Navigation (GAGAN), and Russian System for Differential Correction and Monitoring (SDCM), are in operation. Korea also plans to build the Korea Augmentation Satellite System (KASS), aiming to start operation in 2022 [11].

In some regions, two or more SBAS corrections are accessible with an increase in the number of SBASs. For example, MSAS and SDCM signals are available in Korea. As MSAS provides GPS corrections and SDCM provides GPS and GLONASS corrections, GPS has two corrections from MSAS and SDCM, while GLONASS has one correction from GLONASS [12]. To achieve maximum GPS performance using both MSAS and SDCM, an efficient method to combine both systems is required. However, currently, there is no standard method to integrate and use multi-SBASs [13]. The necessity to combine multi-SBASs and multi-GNSSs will increase as more GNSSs and SBASs are available.

The authors of [13] presented the criteria for selecting proper satellites among multi-SBAS corrections and compared their performance experimentally. The elevation angle, protection level, number of satellites, and degradation of correction information were used as criteria, and the strengths and weaknesses of each criteria were summarized. In [12], the authors proposed an approach to integrate and utilize multi-SBAS corrections. The weighted sum of each SBAS correction was computed and applied as an integrated correction, where the weights were determined using integrity information, such as fast and long-term degradation confidence, user ionospheric range error confidence, airborne receiver error confidence, and tropospheric error confidence. The authors demonstrated that the integration of MSAS and GAGAN in Singapore can improve availability and accuracy. The authors of [14] proposed a more practical method that also considered various situations, such as insufficient correction information in one SBAS. In our previous study [7], the integrated position was obtained from the weighted sum of the positions obtained from each combination of multi-GNSS and multi-SBAS. Since each position was independently computed and combined using arbitrary weights, it had a simple structure that could easily cope with various environments.

As mentioned, a few combination methods were proposed and applied, but their performances were verified in different respective environments, such as the GNSS-used region. To apply the combination methods to navigation systems, their performances should be compared in common environments and through reasonable evaluation methods. Additionally, each method’s characteristics should be analyzed.

This paper presents three efficient methods of combining multi-SBAS corrections in multi-GNSS positioning and compares their performance indices, such as precision, availability, flexibility, and computation complexity, where flexibility implies the ability to implement, modify, and tune easily and simply. The first method, correction domain integration (CDI), was proposed in [12,14]. The second method was measurement domain integration (MDI), proposed in [14]. The third method was position domain integration (PDI), proposed by the authors of [7]. The optimum weights for the three methods were derived and their precisions were compared with covariance analysis. The results of the analysis were verified by an experiment using real measurements and corrections from a commercial GNSS receiver. Comparisons of the computational complexity, the required number of measurements, and ease of application of the three methods were also performed. We thought that the results of the performance comparison and analysis for the various combination methods presented in this paper will be a guideline for the implementation of navigation systems using multiple SBAS and GNSS.

The rest of the paper is organized as follows: Section 2 briefly describes the SBAS operations and provides the covariance analysis. In Section 3, three integration methods (CDI, MDI, and PDI) for single GNSS positioning with multi-SBAS are presented and their covariance analysis is given. Section 4 expands the results of Section 3 to multi-GNSS positioning with multi-SBAS. Section 5 verifies the results using real measurements and corrections. Section 6 summarizes and discusses the properties of each integration method. The final section draws the conclusions.

## 2. Single GNSS Positioning and Error Analysis with Single SBAS Correction

A GNSS positioning procedure with SBAS correction was derived and the performance of the obtained position was evaluated using the covariance analysis. GNSS pseudo-range measurement was denoted as Equation (1), where subscript *i* represents the GNSS type, such as GPS, GLONASS, Galileo, BDS, and so on:

(1) $$\Psi_{i}=r_{i}+cB_{i}+cb_{i}+E_{i}+I_{i}+T_{i}+v_{i}.$$

If $N_{i}$ satellites are available in a GNSS, $\Psi_{i}\in R^{N_{i}\times 1}$ is a pseudo-range measurements vector, and $r_{i}\in R^{N_{i}\times 1}$ is the distance vector between the satellites and a receiver. $cB_{i}$ is the product of a receiver clock bias $B\in R^{1\times 1}$ and speed of light *c*, and $cb_{i}$ is the product of a satellite clock bias vector $b_{i}\in R^{N_{i}\times 1}$ and speed of light. $E_{i}\in R^{N_{i}\times 1}$, $I_{i}\in R^{N_{i}\times 1}$, and $T_{i}\in R^{N_{i}\times 1}$ indicate the ephemeris error vector, ionospheric delay vector, and tropospheric delay vector, respectively. In addition, a receiver measurement noise vector $v_{i}∼N(0,\sigma_{i}^{2}I_{N_{i}})\in R^{N_{i}\times 1}$ is the additive white Gaussian noise (AWGN) with zero mean and ${\left(\sigma_{i}\right)}^{2}$ variance, where $I_{N_{i}}$ is an identity matrix with dimension $N_{i}$.

As a wide-area differential GNSS, an SBAS provides three types of corrections using geostationary satellites to complement and supplement GNSS position accuracy and integrity. First, the fast correction can compensate a range measurement using two parameters: pseudo-range correction (PRC) and range rate correction (RRC). The fast correction parameters are included in Message types (MTs) 2–5 and they are transmitted every 6–60 s to the receiver. Second, the long-term correction eliminates the satellite position and satellite clock error. Because MTs 24 and 25 are transmitted in 120 s periods, they require a relatively longer time than fast correction. Third, ionospheric correction provides the amount of ionospheric delays in the service area using MTs 18 and 26. The receiver can compute the ionospheric delays of the ionospheric pierce point (IPP) by using the surrounding ionospheric grid points (IGPs).

**Figure 1:**
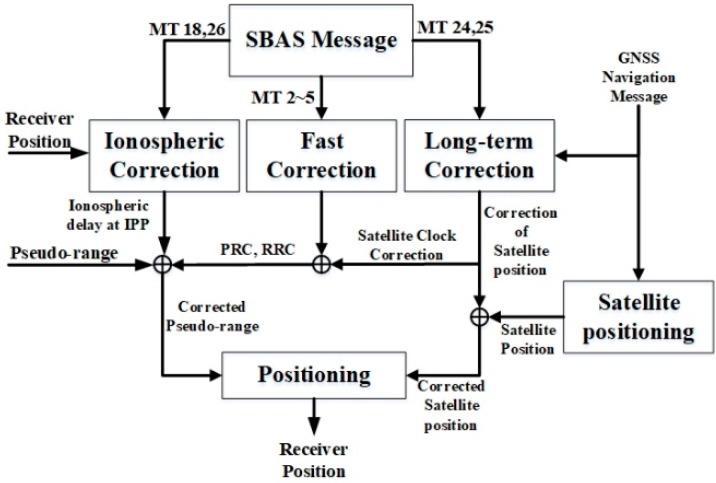
Positioning procedure using satellite-based augmentation system (SBAS) corrections.

Figure 1 shows the positioning procedure using three corrections provided by an SBAS. Using the fast, long-term, and ionospheric corrections, $cb_{i}+E_{i}+I_{i}$ terms in Equation (1) can be compensated. In addition, using the tropospheric corrections in [15] (p. 253), which is not seen in Figure 1 because standard procedures are not defined, the $T_{i}$ term can also be corrected. Therefore, the computed SBAS corrections can be denoted using Equation (2), where the superscript *j* represents the type of SBAS, including WAAS, MSAS, SDCM, EGNOS, and KASS:

(2) $$\eta_{i}^{j}\equiv cb_{i}^{j}+E_{i}^{j}+I_{i}^{j}+T_{i}^{j}.$$

Besides the three corrections, SBAS provides integrity and degradation information for each correction for the *k*th satellite in a GNSS variance of fast correction plus long-term correction ${\left(\sigma_{i,flt}^{j}\right)}_{k}^{2}$ and variance of ionospheric correction ${\left(\sigma_{i,UIRE}^{j}\right)}_{k}^{2}$, where superscript 2 is squared. In addition, the variance of tropospheric delay compensation ${\left(\sigma_{i,tropo}^{j}\right)}_{k}^{2}$ can be computed, as shown in [15] (p. 254).

In this paper, the variance of SBAS correction for the *k*th satellite in a GNSS, ${\left(\sigma_{i}^{j}\right)}_{k}^{2}$, was computed as the sum of the fast, long-term, ionospheric, and tropospheric corrections, as shown in Equation (3), and was adopted as a weighting in the weighted least squares (WLS) positioning algorithm [16,17]:

(3) $${\left(\sigma_{i}^{j}\right)}_{k}^{2}={\left(\sigma_{i,flt}^{j}\right)}_{k}^{2}+{\left(\sigma_{i,UIRE}^{j}\right)}_{k}^{2}+{\left(\sigma_{i,tropo}^{j}\right)}_{k}^{2}.$$

Because each correction of SBAS is uncorrelated, its covariance matrix is denoted as a diagonal matrix and can be written as Equation (4), where *diag* indicates a diagonal matrix and the diagonal terms ${\left(\sigma_{i}^{j}\right)}_{k}^{2},k=1,\ldots ,N_{i}$ can be computed using Equation (3):

(4) $$\mathrm{cov}(\eta_{i}^{j})=diag({\left(\sigma_{i}^{j}\right)}_{1}^{2},{\left(\sigma_{i}^{j}\right)}_{2}^{2},\ldots ,{\left(\sigma_{i}^{j}\right)}_{N_{i}}^{2}).$$

The corrected pseudo-range measurements were obtained using Equation (5) by applying the SBAS corrections of Equation (2) to the pseudo-range of Equation (1). The error $\delta \eta_{i}^{j}=\eta_{i}-\eta_{i}^{j}+v_{i}$ is the sum of receiver noise $v_{i}$ and the remaining terms after applying SBAS corrections $\eta_{i}-\eta_{i}^{j}$; without loss of generality, it is assumed as AWGN with zero mean vector and $R_{i}^{j}$ covariance matrix [16] (p. 301).

(5) $$\Psi_{i}^{j}=\Psi_{i}-\eta_{i}^{j}=r_{i}+cB_{i}+\delta \eta_{i}^{j}\sim N(0,R_{i}^{j}).$$

The covariance of receiver noise can be denoted as a matrix, which consists of the variance of each satellite: ${\left(\sigma_{i}\right)}_{1}^{2}$, ${\left(\sigma_{i}\right)}_{2}^{2}$,…,${\left(\sigma_{i}\right)}_{N_{i}}^{2}$ because the each channel’s measurement noise in receiver can be different based on the elevation angle of satellite. Therefore, the covariance matrix of corrected measurements can be expressed as Equation (6):

(6) $$R_{i}^{j}\equiv \mathrm{cov}(\delta \eta_{i}^{j})=\left[\begin{array}{cccc} {\left(\sigma_{i}^{j}\right)}_{1}^{2} & 0 & & 0\\ 0 & {\left(\sigma_{i}^{j}\right)}_{2}^{2} & & 0\\ & & \ddots & \\ 0 & 0 & & {\left(\sigma_{i}^{j}\right)}_{N_{i}}^{2} \end{array}\right]+\left[\begin{array}{cccc} {\left(\sigma_{i}\right)}_{1}^{2} & 0 & & 0\\ 0 & {\left(\sigma_{i}\right)}_{2}^{2} & & 0\\ & & \ddots & \\ 0 & 0 & & {\left(\sigma_{i}\right)}_{N_{i}}^{2} \end{array}\right].$$

By linearizing at a linearization point, Equation (5) becomes Equation (7), where ${\left[r_{i}^{j}+cB_{i}\right]}_{0}$ is the sum of the computed range and clock bias, $H_{i}^{j}$ is a design matrix, and $\delta x_{i}^{j}$ is the offset of the user’s position and clock bias from the linearization point. As the linearization point is close to the user’s position, a few iterations are required to calculate this position [16]. The linearization point can be initialized by a known position that is close the user’s current or previously calculated position.

(7) $$\delta \Psi_{i}^{j}\equiv \Psi_{i}^{j}-{\left[r_{i}^{j}+cB_{i}\right]}_{0}=H_{i}^{j}\delta x_{i}^{j}+\delta \eta_{i}^{j}.$$

Applying WLS with the weighting of Equation (6), the estimated navigation solution $\delta x_{i}^{j}$ and its covariance are obtained as follows:

(8) $$\delta x_{i}^{j}={\left[{\left(H_{i}^{j}\right)}^{T}{\left(R_{i}^{j}\right)}^{-1}H_{i}^{j}\right]}^{-1}{\left(H_{i}^{j}\right)}^{T}{\left(R_{i}^{j}\right)}^{-1}\delta \Psi_{i}^{j}\;\mathrm{and}$$

(9) $$\mathrm{cov}(\delta x_{i}^{j})={\left[{\left(H_{i}^{j}\right)}^{T}{\left(R_{i}^{j}\right)}^{-1}H_{i}^{j}\right]}^{-1}.$$

In our analysis, $H_{i}^{j}=H_{j}$ is held because the satellites are very far away from the user and the effect of SBAS corrections to the line of sight vector is negligible. If the quality of the pseudo-range is improved by SBAS corrections, the covariance of the corrected pseudo-range measurement $R_{i}^{j}$ will be decreased as compared to that of the uncorrected pseudo-range measurement $R_{i}$. This implies that the precision of the estimated navigation solution $\delta x_{i}^{j}$ is improved by applying SBAS corrections.

## 3. Single GNSS Positioning and Error Analysis with Multi-SBAS Correction

In this section, three methods (CDI, MDI, and PDI) to integrate multi-SBAS corrections for a single GNSS are presented and their performances are compared through a covariance analysis.

### 3.1. CDI

**Figure 2:**
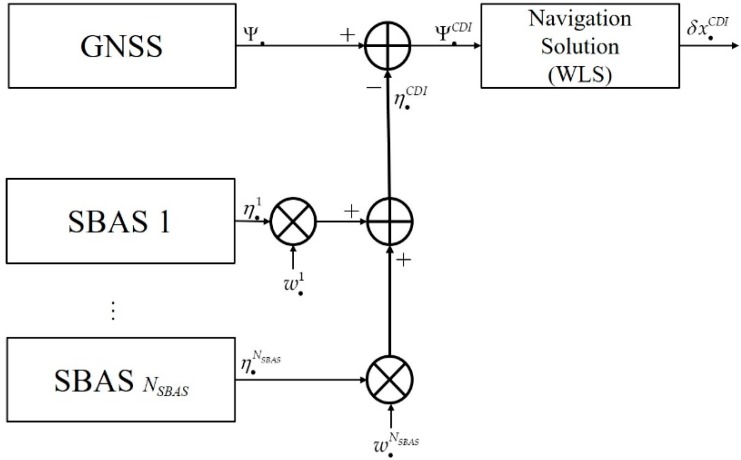
Correction domain integration (CDI) for single global navigation satellite system(GNSS) augmented with multi-SBAS.

The concept of CDI is shown in Figure 2, where $\Psi_{•}$ is a pseudo-range measurement vector, $\eta_{•}^{1},\eta_{•}^{2},\ldots ,\eta_{•}^{N_{SBAS}}$ are the computed correction vectors of multi-SBAS, and $w_{•}^{1},w_{•}^{2},\ldots ,w_{•}^{N_{SBAS}}$ are the weight vectors. In this section, subscript (•), instead of *i*, is used to indicate an unspecific GNSS system. The corresponding corrections are weighted and then summed to generate the CDI correction, as shown in Equation (10). And Equation (11) denotes the corrected pseudo-range.

(10) $$\eta_{•}^{CDI}=w_{•}^{1}\eta_{•}^{1}+\cdots +w_{•}^{N_{SBAS}}\eta_{•}^{N_{SBAS}}=\sum_{j=1}^{N_{SBAS}}w_{•}^{j}\eta_{•}^{j}.$$

(11) $$\Psi_{•}^{CDI}=\Psi_{•}-\eta_{•}^{CDI}=r_{•}+cB_{•}+\delta \eta_{•}^{CDI}\sim N(0,R_{•}^{CDI}).$$

Because the corrections from each SBAS are uncorrelated, the covariance of the corrected pseudo-range $R_{•}^{CDI}$ can be written as

(12) $$R_{•}^{CDI}=w_{•}^{1}R_{•}^{1}{\left(w_{•}^{1}\right)}^{T}+\cdots +w_{•}^{N_{SBAS}}R_{•}^{N_{SBAS}}{\left(w_{•}^{N_{SBAS}}\right)}^{T}=\sum_{j=1}^{N_{SBAS}}w_{•}^{j}R_{•}^{j}{\left(w_{•}^{j}\right)}^{T}.$$

By applying WLS to Equation (11), the navigation solution and covariance of CDI can be derived as Equations (13) and (14), respectively, where $R_{•}^{CDI}$ is utilized as a weight matrix for WLS:

(13) $$\delta x_{•}^{CDI}={\left[{\left(H_{•}^{CDI}\right)}^{T}{\left(R_{•}^{CDI}\right)}^{-1}H_{•}^{CDI}\right]}^{-1}{\left(H_{•}^{CDI}\right)}^{T}{\left(R_{•}^{CDI}\right)}^{-1}\delta \Psi_{•}^{CDI}\;\mathrm{and}$$

(14) $$\mathrm{cov}(\delta x_{•}^{CDI})={\left[{\left(H_{•}^{CDI}\right)}^{T}{\left(R_{•}^{CDI}\right)}^{-1}H_{•}^{CDI}\right]}^{-1}.$$

In Equations (13) and (14), $H_{•}^{CDI}$ becomes $H_{•}$, as mentioned in Section 2, since the satellites are very far away from the user. Using an optimum weighting of Equation (15) [18], Equation (12) becomes Equation (16) by using the fact that the covariance matrix is a diagonal symmetric matrix.

(15) $$w_{•}^{j}={\left[\sum_{m=1}^{N_{SBAS}}{\left(R_{•}^{m}\right)}^{-1}\right]}^{-1}{\left(R_{•}^{j}\right)}^{-1}\;.$$

(16) $$R_{•}^{CDI}=\sum_{j=1}^{N_{SBAS}}\left\{{\left[\sum_{m=1}^{N_{SBAS}}{\left(R_{•}^{m}\right)}^{-1}\right]}^{-1}{\left(R_{•}^{j}\right)}^{-1}R_{•}^{j}{\left(R_{•}^{j}\right)}^{-1}{\left[\sum_{m=1}^{N_{SBAS}}{\left(R_{•}^{m}\right)}^{-1}\right]}^{-1}\right\}={\left[\sum_{j=1}^{N_{SBAS}}{\left(R_{•}^{j}\right)}^{-1}\right]}^{-1}.$$

Because ${\left(R_{•}^{CDI}\right)}^{-1}\delta \Psi_{•}^{CDI}$ is ${\left(R_{•}^{1}\right)}^{-1}\delta \Psi_{•}^{1}+{\left(R_{•}^{2}\right)}^{-1}\delta \Psi_{•}^{2}+\ldots +{\left(R_{•}^{N_{SBAS}}\right)}^{-1}\delta \Psi_{•}^{N_{SBAS}}$, the navigation solution of Equation (13) can be re-written as that of Equation (17). Similarly, the covariance of CDI can be derived as Equation (18).

(17) $$\delta x_{•}^{CDI}={\left[{\left(H_{•}\right)}^{T}\left\{\sum_{j=1}^{N_{SBAS}}{\left(R_{•}^{j}\right)}^{-1}\right\}H_{•}\right]}^{-1}{\left(H_{•}\right)}^{T}\left\{\sum_{j=1}^{N_{SBAS}}{\left(R_{•}^{j}\right)}^{-1}\delta \Psi_{•}^{j}\right\}.$$

(18) $$\mathrm{cov}(\delta x_{•}^{CDI})={\left[{\left(H_{•}\right)}^{T}\sum_{j=1}^{N_{SBAS}}{\left(R_{•}^{j}\right)}^{-1}H_{•}\right]}^{-1}.$$

This result shows that the precision of the navigation solution may depend on the covariance that was expressed as Equation (18). And the covariance will be reduced by the integration of the inverse of each covariance of corrected measurements. As the simplest and best example, if all corrections of SBASs exhibit the same performance, i.e., $R_{•}^{1}=R_{•}^{2}=\ldots =R_{•}^{N_{SBAS}}\equiv R_{•}^{}$, the covariance of Equation (18) becomes ${\left[{\left(H_{•}\right)}^{T}{\left(R_{•}\right)}^{-1}H_{•}\right]}^{-1}/N_{SBAS}$, which means that the precision of the navigation solution is *N_SBAS_* times better than that of a single SBAS.

### 3.2. MDI

**Figure 3:**
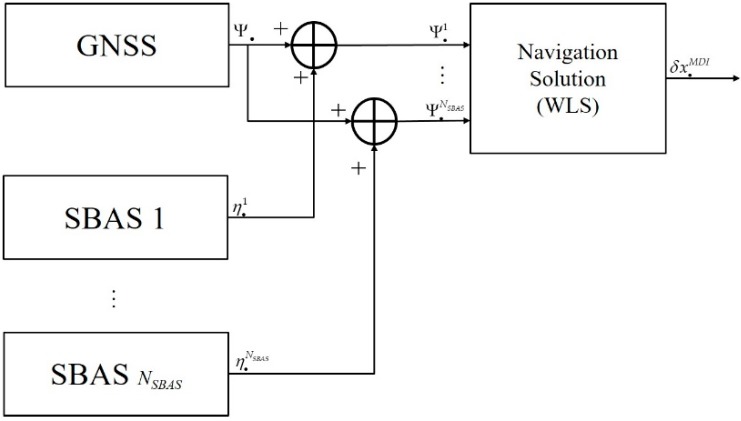
Measurement domain integration (MDI) for single GNSS augmented with multi-SBAS.

The concept of MDI is shown in Figure 3. In MDI, *N_SBAS_* corrections from multi-SBAS were applied to a pseudo-range. Equation (19) is a linearized equation for each corrected pseudo-range and is simply denoted as Equation (20).

(19) $$\left[\begin{array}{c} \delta \Psi_{•}^{1}\\ \vdots \\ \delta \Psi_{•}^{N_{SBAS}} \end{array}\right]=\left[\begin{array}{c} H_{•}^{1}\\ \vdots \\ H_{•}^{N_{SBAS}} \end{array}\right]\delta x_{•}^{MDI}+\left[\begin{array}{c} \delta \eta_{•}^{1}\\ \vdots \\ \delta \eta_{•}^{N_{SBAS}} \end{array}\right]\sim N(\left[\begin{array}{c} 0\\ \vdots \\ 0 \end{array}\right],\left[\begin{array}{ccc} R_{•}^{1} & & 0\\ & \ddots & \\ 0 & & R_{•}^{N_{SBAS}} \end{array}\right]).$$

(20) $$\delta \Psi_{•}^{MDI}\;=\;H_{•}^{MDI}\delta x_{•}^{MDI}+\delta \eta_{•}^{MDI}\sim N(0,R_{•}^{MDI}).$$

By applying WLS to Equation (20), the navigation solution and its covariance are obtained as Equations (21) and (22), respectively. In the derivation, $H_{•}^{1}=H_{•}^{2}\ldots =H_{•}^{N_{SBAS}}\equiv H_{•}$ was used because the satellites are very far away from the user.

(21) $$\begin{array}{cc} \delta x_{•}^{MDI} & ={\left[{\left(H_{•}^{MDI}\right)}^{T}{\left(R_{•}^{MDI}\right)}^{-1}H_{•}^{MDI}\right]}^{-1}{\left(H_{•}^{MDI}\right)}^{T}{\left(R_{•}^{MDI}\right)}^{-1}\delta \Psi_{•}^{MDI}\\ & ={\left[\sum_{j=1}^{N_{SBAS}}{\left(H_{•}^{j}\right)}^{T}{\left(R_{•}^{j}\right)}^{-1}H_{•}^{j}\right]}^{-1}\sum_{j=1}^{N_{SBAS}}{\left(H_{•}^{j}\right)}^{T}{\left(R_{•}^{j}\right)}^{-1}\delta \Psi_{•}^{j}\\ & ={\left[{\left(H_{•}\right)}^{T}\left\{\sum_{j=1}^{N_{SBAS}}{\left(R_{•}^{j}\right)}^{-1}\right\}H_{•}\right]}^{-1}H_{•}^{T}\left\{\sum_{j=1}^{N_{SBAS}}{\left(R_{•}^{j}\right)}^{-1}\delta \Psi_{•}^{j}\right\} \end{array}$$

(22) $$\mathrm{cov}(\delta x_{•}^{MDI})={\left[\sum_{j=1}^{N_{SBAS}}{\left(H_{•}^{j}\right)}^{T}{\left(R_{•}^{j}\right)}^{-1}H_{•}^{j}\right]}^{-1}={\left[{\left(H_{•}\right)}^{T}\sum_{m=1}^{N_{SBAS}}{\left(R_{•}^{m}\right)}^{-1}H_{•}\right]}^{-1}.$$

A comparison of Equations (17) and (18) and Equations (21) and (22) clearly shows that the navigation solution and the covariance of MDI and CDI are identical.

### 3.3. PDI

**Figure 4:**
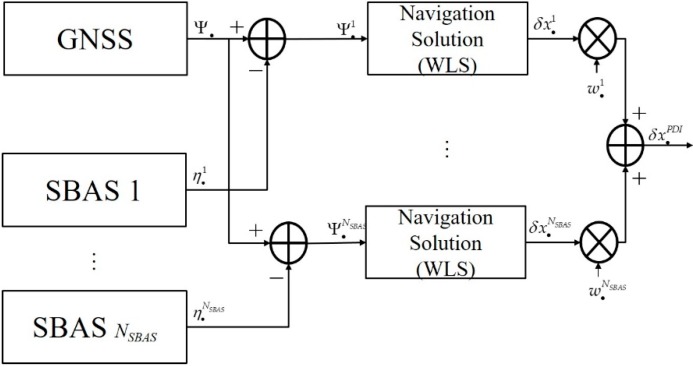
Position domain integration (PDI) for single GNSS augmented with multi-SBAS.

In the PDI, shown in Figure 4, first, *N_SBAS_* navigation solutions were computed independently using the *N_SBAS_*-corrected pseudo-range. The procedure for finding each navigation solution is explained in Section 2. Then, *N_SBAS_* navigation solutions were weighted-summed to obtain the navigation solution of the PDI $\delta x_{•}^{PDI}$, as shown in Equation (23) with covariance of Equation (24).

(23) $$\delta x_{•}^{PDI}=\sum_{j=1}^{N_{SBAS}}w_{•}^{j}\delta x_{•}^{j}.$$

(24) $$\mathrm{cov}(\delta x_{•}^{PDI})=\sum_{j=1}^{N_{SBAS}}w_{•}^{j}{\left[{\left(H_{•}^{j}\right)}^{T}{\left(R_{•}^{j}\right)}^{-1}H_{•}^{j}\right]}^{-1}{\left(w_{•}^{j}\right)}^{T}.$$

Using Equation (25) as an optimum weighting [18], Equations (23) and (24) become Equations (26) and (27), respectively.

(25) $$w_{•}^{j}={\left[\sum_{m=1}^{N_{SBAS}}\left\{{\left(H_{•}^{m}\right)}^{T}{\left(R_{•}^{m}\right)}^{-1}H_{•}^{m}\right\}\right]}^{-1}{\left(H_{•}^{j}\right)}^{T}{\left(R_{•}^{j}\right)}^{-1}H_{•}^{j}.$$

(26) $$\delta x_{•}^{PDI}={\left[\sum_{j=1}^{N_{SBAS}}{\left(H_{•}^{j}\right)}^{T}{\left(R_{•}^{j}\right)}^{-1}H_{•}^{j}\right]}^{-1}\sum_{j=1}^{N_{SBAS}}{\left(H_{•}^{j}\right)}^{T}{\left(R_{•}^{j}\right)}^{-1}\delta \Psi_{•}^{j}={\left[{\left(H_{•}\right)}^{T}\sum_{j=1}^{N_{SBAS}}{\left(R_{•}^{j}\right)}^{-1}H_{•}\right]}^{-1}{\left(H_{•}\right)}^{T}\sum_{j=1}^{m}{\left(R_{•}^{j}\right)}^{-1}\delta \Psi_{•}^{j}.$$

(27) $$\mathrm{cov}(\delta x_{•}^{PDI})={\left[\sum_{j=1}^{N_{SBAS}}{\left(H_{•}^{j}\right)}^{T}{\left(R_{•}^{j}\right)}^{-1}H_{•}^{j}\right]}^{-1}={\left[{\left(H_{•}\right)}^{T}\sum_{j=1}^{N_{SBAS}}{\left(R_{•}^{j}\right)}^{-1}H_{•}\right]}^{-1}.$$

Equations (26) and (27) show that the navigation solution and covariance of PDI are same as those of CDI and MDI. This means that the three combination methods (CDI, MDI, and PDI) provide the same navigation solution with the same quality if the optimum weightings are used in case of a single GNSS and multi-SBAS.

## 4. Multi-GNSS Positioning and Error Analysis with Multi-SBAS Corrections

In this section, the results of single GNSS positioning with multi-SBAS corrections, explained in the previous section, are expanded to multi-GNSS positioning, including GPS, GLONASS, BDS, and Galileo. The CDI, MDI, and PDI were expanded to multi-GNSS and their performances were compared using a covariance analysis.

### 4.1. CDI

**Figure 5:**
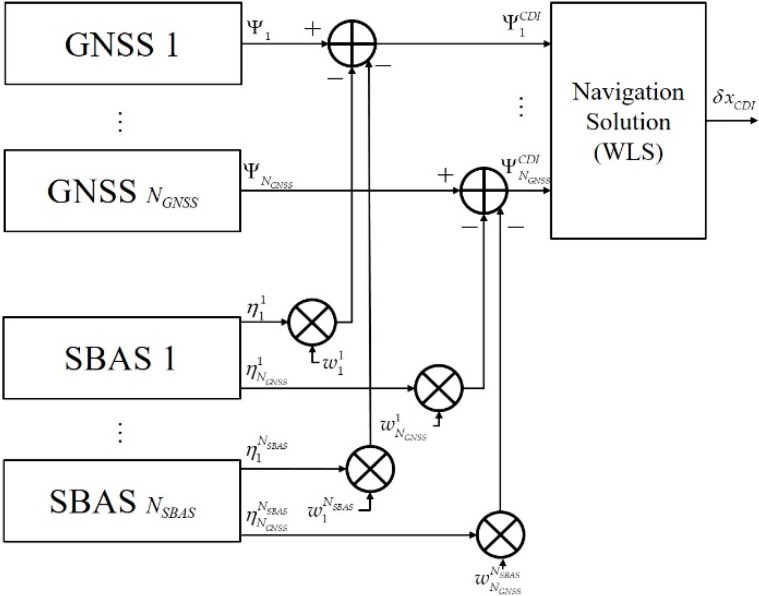
CDI for multi-GNSS augmented with multi-SBAS.

The concept of CDI for *N_GNSS_* GNSS and *N_SBAS_* SBAS is shown in Figure 5. By expanding Equation (10), the weighted sum of corrections for the *i*th GNSS is obtained as:

(28) $$\eta_{i}^{CDI}=\sum_{j=1}^{N_{SBAS}}w_{i}^{j}\eta_{i}^{j},\;i=1,\ldots ,N_{GNSS}.$$

Equations (29) and (30) are the corrected pseudo-range based on CDI and its covariance, respectively:

(29) $$\Psi_{i}^{CDI}=\Psi_{i}-\eta_{i}^{CDI}=r_{i}+cB_{i}+\delta \eta_{i}^{CDI}\sim N(0,R_{i}^{CDI}),$$

(30) $$R_{i}^{CDI}=\sum_{j=1}^{N_{SBAS}}w_{i}^{j}R_{i}^{j}{\left(w_{i}^{j}\right)}^{T},\;i=1,\ldots ,N_{GNSS}.$$

By applying optimal weighting [18] of Equation (31), Equation (30) become Equation (32)

(31) $$w_{i}^{j}={\left[\sum_{j=1}^{N_{SBAS}}{\left(R_{i}^{j}\right)}^{-1}\right]}^{-1}{\left(R_{i}^{j}\right)}^{-1},\;i=1,\ldots ,N_{GNSS},\;j=1,\ldots ,N_{SBAS}.$$

(32) $$R_{i}^{CDI}=\sum_{j=1}^{N_{SBAS}}\left\{{\left[\sum_{m=1}^{N_{SBAS}}{\left(R_{i}^{m}\right)}^{-1}\right]}^{-1}{\left(R_{i}^{j}\right)}^{-1}R_{i}^{j}{\left(R_{i}^{j}\right)}^{-1}{\left[\sum_{m=1}^{N_{SBAS}}{\left(R_{i}^{m}\right)}^{-1}\right]}^{-1}\right\}={\left[\sum_{j=1}^{N_{SBAS}}{\left(R_{i}^{j}\right)}^{-1}\right]}^{-1},\;i=1,\ldots ,N_{GNSS}.$$

By applying WLS to Equation (29) with the weighting of Equation (31), the estimated navigation solution and its covariance are derived as Equations (33) and (34), respectively. During the derivation, $E[\delta \Psi_{i}^{CDI}{\left(\delta \Psi_{j}^{CDI}\right)}^{T}]=0,\;i\neq j$ is used because each $\delta \Psi_{j}^{CDI}$ is uncorrelated.

(33) $$\delta x_{CDI}={\left[\sum_{i=1}^{N_{GNSS}}H_{i}^{T}{\left(R_{i}^{CDI}\right)}^{-1}H_{i}^{}\right]}^{-1}\sum_{i=1}^{N_{GNSS}}H_{i}^{T}{\left(R_{i}^{CDI}\right)}^{-1}\delta \Psi_{i,CDI}^{}={\left[\sum_{i=1}^{N_{GNSS}}H_{i}^{T}\left\{\sum_{j=1}^{N_{SBAS}}{\left(R_{i}^{j}\right)}^{-1}\right\}H_{i}\right]}^{-1}\left[\sum_{i=1}^{N_{GNSS}}H_{i}^{T}\left\{\sum_{j=1}^{N_{SBAS}}{\left(R_{i}^{j}\right)}^{-1}\delta \Psi_{i,j}^{}\right\}\right].$$

(34) $$\mathrm{cov}(\delta x_{CDI}^{})={\left[\sum_{i=1}^{N_{GNSS}}H_{i}^{T}{\left(R_{i}^{CDI}\right)}^{-1}H_{i}^{}\right]}^{-1}={\left[\sum_{i=1}^{N_{GNSS}}H_{i}^{T}\left\{\sum_{j=1}^{N_{SBAS}}{\left(R_{i}^{j}\right)}^{-1}\right\}H_{i}\right]}^{-1}.$$

Equation (34) also shows that, by combining the covariance of correlated measurements, the overall combined covariance can be reduced; it is similar in form to Equation (18). For example, if the quality of each SBAS is equal (i.e., $R_{i}^{1}=\ldots =R_{i}^{N_{SBAS}}$, $i=1,\ldots ,N_{GNSS}$), the covariance will be reduced to 1/*N_SBAS_* and then *N_SBAS_* times performance improvement can be expected. In addition, even if not realistic, if the constellation and number of satellites of each GNSS are the same, i.e., $H_{1}=H_{2}=\ldots =H_{N_{GN\text{​}SS}}$ is satisfied, the covariance is reduced to 1/(*N_SBAS_* × *N_GNSS_*). This implies a (*N_SBAS_* × *N_GNSS_*) times improvement in the precision of the navigation solution is possible by combining the multi-GNSS and multi-SBAS in the CDI scheme.

### 4.2. MDI

**Figure 6:**
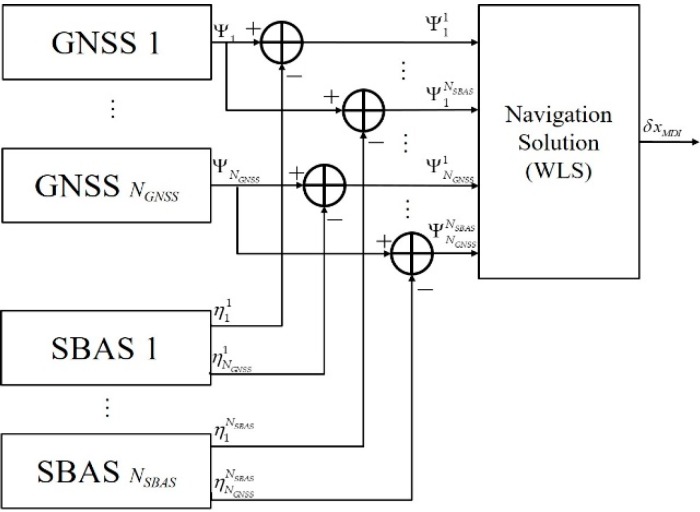
MDI for multi-GNSS augmented with multi-SBAS.

The concept of MDI for *N_GNSS_* GNSS and *N_SBAS_* SBAS is shown in Figure 6. The augmented form of the linearized pseudo-range is denoted as:

(35) $$\left[\begin{array}{c} \delta \Psi_{1}^{1}\\ \delta \Psi_{1}^{2}\\ \vdots \\ \delta \Psi_{1}^{N_{SBAS}}\\ \vdots \\ \delta \Psi_{N_{GNSS}}^{1}\\ \delta \Psi_{N_{GNSS}}^{2}\\ \vdots \\ \delta \Psi_{N_{GNSS}}^{N_{SBAS}} \end{array}\right]=\left[\begin{array}{c} H_{1}^{1}\\ H_{1}^{2}\\ \vdots \\ H_{1}^{N_{SBAS}}\\ \vdots \\ H_{N_{GNSS}}^{1}\\ H_{N_{GNSS}}^{2}\\ \vdots \\ H_{N_{GNSS}}^{N_{SBAS}} \end{array}\right]\delta x_{MDI}+\left[\begin{array}{c} \delta \eta_{1}^{1}\\ \delta \eta_{1}^{2}\\ \vdots \\ \delta \eta_{1}^{N_{SBAS}}\\ \vdots \\ \delta \eta_{N_{GNSS}}^{1}\\ \delta \eta_{N_{GNSS}}^{2}\\ \vdots \\ \delta \eta_{N_{GNSS}}^{N_{SBAS}} \end{array}\right]\sim N(\left[\begin{array}{c} 0\\ 0\\ \vdots \\ 0\\ \vdots \\ 0\\ 0\\ \vdots \\ 0 \end{array}\right],\left[\begin{array}{ccccccccc} R_{1}^{1} & 0 & & 0 & & & & & \\ 0 & R_{1}^{2} & & 0 & & & & & \\ & & \ddots & & & & & & \\ 0 & 0 & & R_{1}^{N_{SBAS}} & & & & & \\ & & & & \ddots & & & & \\ & & & & & R_{N_{GNSS}}^{1} & 0 & & 0\\ & & & & & 0 & R_{N_{GNSS}}^{2} & & 0\\ & & & & & & & \ddots & \\ & & & & & 0 & 0 & & R_{N_{GNSS}}^{N_{SBAS}} \end{array}\right]).$$

By applying WLS to Equation (35), the estimated navigation solution and covariance are obtained as Equations (36) and (37), respectively. Here, $H_{i}^{1}=\ldots =H_{i}^{N_{SBAS}}\equiv H_{i}^{}$ is used again because the satellites are very far away from the user.

(36) $$\begin{array}{cc} \delta x_{MDI} & ={\left[\sum_{i=1}^{N_{GNSS}}\left\{\sum_{j=1}^{N_{SBAS}}{\left(H_{i}^{j}\right)}^{T}{\left(R_{i}^{j}\right)}^{-1}H_{i}^{j}\right\}\right]}^{-1}\left[\sum_{i=1}^{N_{GNSS}}\left\{\sum_{j=1}^{N_{SBAS}}{\left(H_{i}^{j}\right)}^{T}{\left(R_{i}^{j}\right)}^{-1}\delta \Psi_{i}^{j}\right\}\right]\\ & ={\left[\sum_{i=1}^{N_{GNSS}}{\left(H_{i}\right)}^{T}\left\{\sum_{j=1}^{N_{SBAS}}{\left(R_{i}^{j}\right)}^{-1}\right\}H_{i}\right]}^{-1}\left[\sum_{i=1}^{N_{GNSS}}{\left(H_{i}\right)}^{T}\left\{\sum_{j=1}^{N_{SBAS}}(R_{i}^{j}{)}^{-1}\delta \Psi_{i}^{j}\right\}\right] \end{array}$$

(37) $$\mathrm{cov}(\delta x_{MDI})={\left[\sum_{i=1}^{N_{GNSS}}\left\{\sum_{j=1}^{N_{SBAS}}{\left(H_{i}^{j}\right)}^{T}{\left(R_{i}^{j}\right)}^{-1}H_{i}^{j}\right\}\right]}^{-1}={\left[\sum_{i=1}^{N_{GNSS}}{\left(H_{i}\right)}^{T}\left\{\sum_{j=1}^{N_{SBAS}}{\left(R_{i}^{j}\right)}^{-1}\right\}H_{i}\right]}^{-1}.$$

A comparison of Equations (36) and (37) and Equations (33) and (34) clearly shows that the navigation solution and the covariance of MDI and CDI are identical in multi-GNSS as well as in multi-SBAS.

### 4.3. PDI

**Figure 7:**
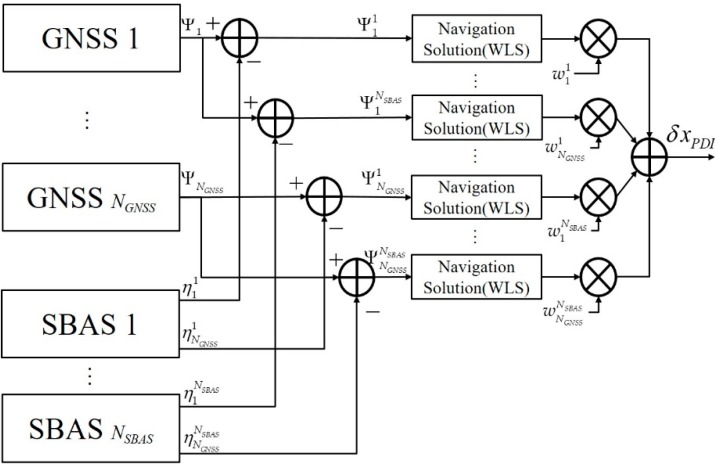
PDI for multi-GNSS augmented with multi-SBAS.

The concept of PDI for *N_GNSS_* GNSS and *N_SBAS_* SBAS is shown in Figure 7. By applying WLS to all corrected pseudo-ranges, (*N_SBAS_* × *N_GNSS_*)-estimated navigation solutions and their covariances are given as Equations (38) and (39), respectively.

(38) $$\delta x_{i}^{j}={\left[{\left(H_{i}^{j}\right)}^{T}{\left(R_{i}^{j}\right)}^{-1}H_{i}^{j}\right]}^{-1}{\left(H_{i}^{j}\right)}^{T}{\left(R_{i}^{j}\right)}^{-1}\delta \Psi_{i}^{j},\;i=1,\ldots ,N_{GNSS},\;j=1,\ldots ,N_{SBAS}.$$

(39) $$\mathrm{cov}(\delta x_{i}^{j})={\left[{\left(H_{i}^{j}\right)}^{T}{\left(R_{i}^{j}\right)}^{-1}H_{i}^{j}\right]}^{-1},\;i=1,\ldots ,N_{GNSS},\;j=1,\ldots ,N_{SBAS}.$$

By expanding Equations (23) and (24), Equations (40) and (41) indicate the PDI navigation solution and covariance, respectively.

(40) $$\delta x_{PDI}=\sum_{i=1}^{N_{GNSS}}\sum_{j=1}^{N_{SBAS}}w_{i}^{j}\delta x_{i}^{j}.$$

(41) $$\mathrm{cov}(\delta x_{PDI})=\sum_{i=1}^{N_{GNSS}}\sum_{j=1}^{N_{SBAS}}w_{i}^{j}{\left[{\left(H_{i}^{j}\right)}^{T}{\left(R_{i}^{j}\right)}^{-1}H_{i}^{j}\right]}^{-1}{\left(w_{i}^{j}\right)}^{T}.$$

Using an optimum weighting [18] of Equation (42), Equations (40) and (41) become Equations (43) and (44), respectively.

(42) $$w_{i}^{j}={\left[\sum_{n=1}^{N_{GNSS}}\sum_{m=1}^{N_{SBAS}}{\left(H_{n}^{m}\right)}^{T}{\left(R_{n}^{m}\right)}^{-1}H_{n}^{m}\right]}^{-1}\left[{\left(H_{i}^{j}\right)}^{T}{\left(R_{i}^{j}\right)}^{-1}H_{i}^{j}\right],\;i=1,\ldots ,N_{GNSS},\;j=1,\ldots ,N_{SBAS}.$$

(43) $$\begin{array}{cc} \delta x_{PDI} & ={\left[\sum_{i=1}^{N_{GNSS}}\sum_{j=1}^{N_{SBAS}}{\left(H_{i}^{j}\right)}^{T}{\left(R_{i}^{j}\right)}^{-1}H_{i}^{j}\right]}^{-1}\sum_{i=1}^{N_{GNSS}}\sum_{j=1}^{N_{SBAS}}{\left(H_{i}^{j}\right)}^{T}{\left(R_{i}^{j}\right)}^{-1}\delta \Psi_{i}^{j}\\ & ={\left[\sum_{i=1}^{N_{GNSS}}{\left(H_{i}^{}\right)}^{T}\left\{\sum_{j=1}^{N_{SBAS}}{\left(R_{i}^{}\right)}^{-1}\right\}H_{i}^{}\right]}^{-1}\left[\sum_{i=1}^{N_{GNSS}}{\left(H_{i}\right)}^{T}\left\{\sum_{j=1}^{N_{SBAS}}{\left(R_{i}^{j}\right)}^{-1}\delta \Psi_{i}^{j}\right\}\right] \end{array}$$

(44) $$\mathrm{cov}(\delta x_{PDI})={\left[\sum_{i=1}^{N_{GNSS}}\sum_{j=1}^{N_{SBAS}}{\left(H_{i}^{j}\right)}^{T}{\left(R_{i}^{j}\right)}^{-1}H_{i}^{j}\right]}^{-1}={\left[\sum_{i=1}^{N_{GNSS}}{\left(H_{i}^{}\right)}^{T}\left\{\sum_{j=1}^{N_{SBAS}}{\left(R_{i}^{j}\right)}^{-1}\right\}H_{i}^{}\right]}^{-1}.$$

Equations (43) and (44) show that the navigation solution and covariance of PDI are the same as those of CDI and MDI. This means that the three methods (CDI, MDI, and PDI) give the same navigation solution with the same quality if the optimum weightings are used in cases of multi-GNSS and multi-SBAS.

## 5. Experiment and Verification Using Real Measurement and Corrections

To verify the analysis results, the navigation solutions of three methods (CDI, MDI, and PDI) that were calculated using real measurements and corrections were compared. In Chungbuk National University in Korea, the real measurements and corrections for 1000 s were collected using a commercial receiver (M8T, u-blox, Thalwil, Switzerland)) that supports receiving GPS L1 C/A, GLONASS G1 C/A, MSAS, and SDCM. Although Korea is not an included service area for MSAS and SDCM, these signals can be received and the given corrections are reasonable. Currently, MSAS provides corrections for only GPS and SDCM provides corrections for both GPS and GLONASS. Therefore, three types of corrected measurements were available, such as GPS corrected by MSAS, GPS corrected by SDCM, and GLONASS corrected by SDCM.

The commercial receiver that was used generated a local time that was estimated by considering the time offset among the different satellite systems. Because the pseudo-range of each GNSS is measured based on the local time, the user does not need to consider the time offset alignment for positioning using multi-GNSS. Therefore, the measurements and corrections from the commercial receiver could be applied to the three methods mentioned above without additional modification. In the collected real measurements, eight possible GPS measurements could be corrected by MSAS. Additionally, six GPS and five GLONASS measurements that could be corrected by SDCM were received.

Using the collected real measurements, navigation solutions were calculated using the three presented methods of CDI, MDI, and PDI. The experiment was performed with two scenarios. The first was where only GPS measurements and multiple corrections were used. The second was where both GPS and GLONASS measurements were used with two types of corrections. By comparing the results of the two scenarios, the efficiency of the expansion to the multi-GNSS that is presented in this paper was demonstrated.

**Figure 8:**
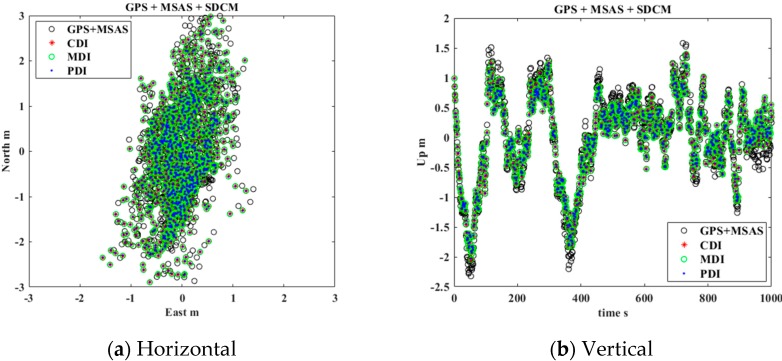
Estimated position errors using CDI, MDI, and PDI for single GNSS and multi-SBAS.

**Figure 9:**
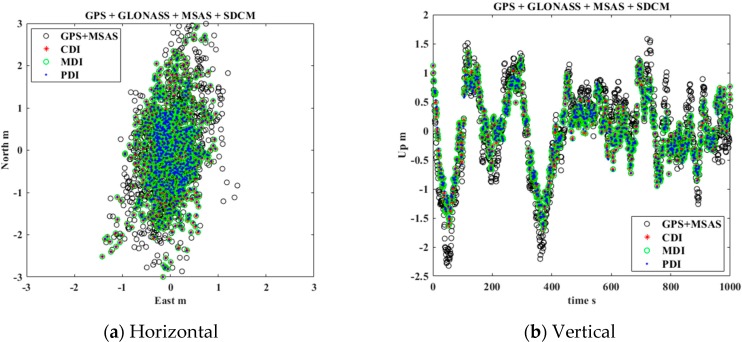
Estimated position errors using CDI, MDI, and PDI for multi-GNSS and multi-SBAS

In general, the reasonable covariance of GNSS and SBAS must be estimated to obtain a more integrated, precise, and accurate position. However, because this experiment focused on comparing the performance of the three integration methods in the same situation, each GNSS and SBAS covariance was initialized as 1.0 m. Subsequently, the positioning results of each method were compared as point-by-point to verify the error analysis results that the precision of the three methods would be equal. The positioning results for the vertical and horizontal errors were presented as east-north-up (ENU) coordinates, as depicted in Figure 8 and Figure 9.

Figure 8a,b depicts the horizontal and vertical errors for GPS, MSAS, and SDCM integration using CDI, MDI, and PDI for single GNSS and multi-SBAS that were mentioned in Section 3. To present the efficiency of multi-SBAS integration, results of the positioning using corrected GPS by MSAS are presented together as black circles. Table 1 presents the standard deviations for the east, north, and up axes that indicate the precision. As can be seen from the experiment results, when CDI, MDI, and PDI were applied, precision improved by approximately 0.01–0.07 m than in the case of GPS with MSAS. Moreover, in the scatter plot of Figure 8, red stars (CDI), green circles (MDI), and blue points (PDI) occur in the same positions. The cm-level precisions of CDI, MDI, and PDI were equal. These results verified that the integration of MSAS and SDCM is more precise than when only one SBAS was used. Additionally, the three integration methods had equal precisions. The experimental results are consistent with the error analysis results of Section 3.

Figure 9a,b depicts the horizontal and vertical errors for GPS, GLONASS, MSAS, and SDCM integration using CDI, MDI, and PDI that were discussed in Section 4 to combine multi-GNSS and multi-SBAS. Additionally, the standard deviations for each axis are presented in Table 2. By adding GLONASS and the SDCM corrections, the standard deviations of each axis decreased by 0.09–0.12 m more than in the case of GPS and MSAS. The experimental results also demonstrated that the cm-level precisions of CDI, MDI, and PDI were measured equally. Therefore, the error analysis result that the three methods for multi-GNSS and multi-SBAS have same precision is reasonable.

## 6. Summary and Discussion

Besides positioning precision, issues such as computational complexity, availability, and flexibility are also important. In this section, a brief analysis of performance, including implementation issues, is given. Table 3 summarizes the results. As shown in Section 3 and Section 4, the covariance analysis indicated that the three methods were the same in precision. However, they differed in computational complexity, availability, and flexibility.

To compare computational complexity, a number of operations, such as addition, multiplication, and division, to obtain a position were analyzed and the results are summarized in Table 4. The number of operations were determined by the number of GNSSs (*m = N_GNSS_)*, the number of SBASs *(n = N_SBAS_)*, and the number of visible satellites. For ease of comparison, the number of visible satellites of each GNSS was assumed to be the same (*l = N*_1_ *= N*_2_ *= … = N_N_GNSS__*).

In CDI, the dimension of the *H* matrix and the number of inverse operations are independent of the number of SBASs, so just a simple operation was added to correct the weighting. By contrast, MDI complexity depends on the number of GNSSs, SBASs, and visible satellites. When multi-GNSS and multi-SBAS are concerned, MDI requires more operations. The PDI requires *m* times (4 × 4) matrix inversion, while CDI and MDI require one. However, the dimension of *H* matrix of MDI is *nml* and it is larger than that of PDI(*l*) and CDI(*l*). The numerical example of 3 GNSSs and 3 SBASs with 8 visible satellites shows that MDI requires more computational loads.

In GNSS, at least four pseudo-range measurements are required to find position and time. In PDI, each GNSS requires at least four visible satellites, while in CDI and MDI, the position can be found with four satellites regardless of GNSS type. This implies that the availability of PDI is worse than the other methods.

As PDI combines the navigation solutions, it can be easily implemented by combining two or more off-the-shelf receivers that support different GNSSs and SBASs. However, MDI and CDI require a modification of off-the-shelf receivers to integrate the corrections and pseudo-range measurements, such as system time difference correction. This means that PDI is more flexible than CDI and MDI in implementation and expansion.

In summary, CDI, MDI, and PDI have the same precision, but each method has different pros and cons of implementation. Although CDI has less computational load, it is less flexible. Therefore, CDI is suitable for a large number of GNSSs and SBASs. MDI is preferred in urban areas where lots of GNSS and SBAS signals are blocked because of high availability, even though it requires more computing resources. PDI is advantageous in implementation since it does not require modification of existing receivers, even though it has a lack of availability. As more GNSSs and more SBASs are accessible in near future, the ease of implementation might be more important in combing multi-SBAS corrections in multi-GNSS.

## 7. Conclusions

Currently, multi-SBAS utilization methods are not officially provided. Although a few methods have already been proposed, each method was verified in different environments and their pros and cons were not presented. In this study, to effectively utilize multi-SBAS corrections in multi-GNSS positioning, three methods, namely, CDI, MDI, and PDI, were presented and their performances were compared. The structures and navigation algorithms for single GNSS with multi-SBAS corrections were presented and then the results were expanded to multi-GNSS. The optimum weights were derived from covariance analysis and, when the optimum weightings of the three methods were applied to the WLS, the analysis revealed that the precisions of the three methods are equal. Experimental results using a commercial receiver and real measurements and corrections confirmed the analysis. Besides precision, implementation issues, such as computational complexity, availability, and flexibility, were also analyzed. CDI requires a small computational load, but is less flexible. MDI provides high availability with more computational complexity. PDI is more flexible, but has less availability. The results are expected to be a useful guideline in the design, implementation, and analysis of an efficient multi-GNSS positioning system using multi-SBAS corrections, which will be common in applications in the near future.

## Figures and Tables

**Table 1:** Standard deviation of CDI, MDI, and PDI for single GNSS and multi-SBAS integration.

Standard Deviation [m]	East	North	Up
GPS with MSAS	0.45	1.16	0.78
CDI	0.44	1.15	0.71
MDI	0.44	1.15	0.71
PDI	0.44	1.15	0.71

**Table 2:** Standard deviation of CDI, MDI, and PDI for multi-GNSS and multi-SBAS integration.

Standard Deviation [m]	East	North	Up
GPS with MSAS	0.45	1.16	0.78
CDI	0.36	1.05	0.66
MDI	0.36	1.05	0.66
PDI	0.36	1.05	0.66

**Table 3:** Comparison of CDI, MDI, and PDI.

	CDI	MDI	PDI
Precision	${\left[\sum_{i=1}^{N_{GNSS}}{\left(H_{i}\right)}^{T}\left\{\sum_{j=1}^{N_{SBAS}}{\left(R_{i}^{j}\right)}^{-1}\right\}H_{i}\right]}^{-1}$	${\left[\sum_{i=1}^{N_{GNSS}}{\left(H_{i}\right)}^{T}\left\{\sum_{j=1}^{N_{SBAS}}{\left(R_{i}^{j}\right)}^{-1}\right\}H_{i}\right]}^{-1}$	${\left[\sum_{i=1}^{N_{GNSS}}{\left(H_{i}\right)}^{T}\left\{\sum_{j=1}^{N_{SBAS}}{\left(R_{i}^{j}\right)}^{-1}\right\}H_{i}\right]}^{-1}$
Complexity	Low	High	Middle
Availability	$\sum_{i=1}^{N_{GNSS}}N_{i}\geq 4$	$\sum_{i=1}^{N_{GNSS}}N_{i}\geq 4$	$N_{i}\geq 4,\;i=1,\ldots ,N_{GNSS}$
Flexibility	Low	Low	High

**Table 4:** Complexity of CDI, MDI, and PDI.

	CDI	MDI	PDI
Number of additions	$8l^{2}m+nml+mn-5m+135$	$8{\left(nml\right)}^{2}+27nml+83$	$12l^{2}nm+39nml+95nm+75$
Number of multiplications	$8l^{2}m+nml+24ml+424$	$8{\left(nml\right)}^{2}+39nml+360$	$12l^{2}nm+55nml+376nm+360$
Number of divisions	nml+3ml+m+16	4nml+16	4nml+16nm
Total number of operations (n=3,m=3,l=8)	4508	88443	25698
